# Apology

**Published:** 1852-05

**Authors:** 


*Apology.*

WE regret that on the occasion of our first introduction to our readers, we are obliged to appear under such an awkward caption; but "time and chance happeneth to all men." The new type promised in the prospectus for the present volume was ordered early in March, but, for. unforeseen reasons, was delayed five or six weeks. Our printers insisted on a new dress, and the result is, that the present number will reach our subscribers about three weeks after the proper time. We hope, however, the above will be considered a sufficient excuse. Hereafter we will be punctual in our monthly visits—to those of our friends who will set the example by being prompt in their remittances.

